# Postoperative decrease of albumin (ΔAlb) as early predictor of complications after gastrointestinal surgery: a systematic review

**DOI:** 10.1186/s13741-022-00238-3

**Published:** 2022-02-15

**Authors:** Gaëtan-Romain Joliat, Arnaud Schoor, Markus Schäfer, Nicolas Demartines, Martin Hübner, Ismail Labgaa

**Affiliations:** 1 Department of Visceral Surgery, Lausanne University Hospital (CHUV), University of Lausanne (UNIL), Rue du Bugnon 46, CH-1011 Lausanne, Switzerland; 2 Graduate School of Health Sciences, University of Bern, Bern, Switzerland; 3 Department of General and Visceral Surgery, HIB Hospital, Payerne, Switzerland

**Keywords:** Complications, Predictor, ΔAlb, Biomarkers

## Abstract

**Background:**

Postoperative complications are frequent after gastrointestinal surgery and early prediction remains an unmet need. Serum albumin shows a rapid decrease after surgery, and this decline (ΔAlb) may reflect the intensity of the surgical stress response and thereby be a predictor of postoperative complications. This study aimed to comprehensively review the available data on ΔAlb in gastrointestinal surgery.

**Methods:**

PRISMA guidelines were followed to conduct a systematic review of the literature in MEDLINE and Embase. Studies assessing the role of ΔAlb to predict complications after gastrointestinal surgery were included.

**Results:**

A total of 1256 articles were screened, and 16 studies were included in the final analysis: 7 prospective and 9 retrospective trials. Sensitivity of ΔAlb to predict postoperative complications ranged from 63 to 84%, whereas specificity ranged from 61 to 86%. Nine out of the 16 included studies established a threshold of ΔAlb to predict morbidity (range: 5–11 g/l or 14–27%).

**Conclusion:**

ΔAlb appeared as a valuable and promising biomarker to anticipate complications after gastrointestinal surgery. Future efforts are needed to determine whether and how ΔAlb may be integrated in clinical practice to guide clinicians in the perioperative management of patients.

## Background

Gastrointestinal (GI) surgery is associated with a substantial risk of postoperative complications, reported in almost 50% of cases after major operations (Jarnagin et al., 2002). Not only detrimental to patients, they also induce a significant increase in costs, a timely concern while healthcare expenditures particularly need to be controlled (Straatman et al., 2015; Vonlanthen et al., 2011). It is important for surgeons to identify preoperatively patients at risk (e.g., patients with pre-existing cardiopulmonary pathology or with malnutrition) and to rapidly recognize patients who will develop postoperative complications. Being the first step to anticipate complications, early identification of patients at risk is a *sine qua non* condition to succeed. To this regard, available biomarkers such as white blood cells count, C-reactive protein (CRP), procalcitonin, or interleukin-6 are quite disappointing, being poorly accurate, with low sensitivity or specificity, with slow kinetics, relatively late indicators, expensive, or difficultly reproducible (Labgaa et al., 2017a). An example of a biomarker that changed the management is the negative predictive value of CRP after colorectal surgery. A meta-analysis showed that discharge based on the value of CRP was safe after colorectal surgery (Warschkow et al., 2012). Another example is the realization of CT scan based on postoperative CRP values. This strategy has been shown in colorectal surgery to detect postoperative complications earlier (McSorley et al., 2017).

Albumin is a negative acute phase protein with fast decline in case of inflammation. This phenomenon is mainly caused by redistribution into the third space and was observed already during the first hours after various types of surgical procedures (Labgaa et al., 2017b). Moreover, the degree of albumin decrease was commensurate with the surgical trauma. In addition, surgical trauma (extent of surgery) was associated with stress response which is also associated with postoperative complications (Thorell et al., 1999). In that context, the amplitude of albumin decrease was referred as ΔAlb and recent studies investigated whether it could be used as a predictor of postoperative complications. In addition of displaying attractive characteristics such as being performant, early indicative, easy to measure, and inexpensive, these studies revealed promising data on its predictive value for postoperative complications (Labgaa et al., 2017b). Enhanced Recovery After Surgery (ERAS) is a multimodal perioperative management pathway with the goal of improving postoperative recovery by decreasing the response to surgical stress. ERAS via goal-directed fluid therapy or rapid onset of oral nutrition might have an influence on ΔAlb that reflects the magnitude of the inflammatory response to the surgical trauma. In liver surgery, ERAS has been shown to decrease ΔAlb compared to non-ERAS patients (median ΔAlb: 12 *vs*. 16 g/l, *p*< 0.001) (Gonvers et al., 2021).

The present study aimed to perform a systematic review of available data on ΔAlb in GI surgery.

## Methods

### Search

A systematic review of the literature was performed using MEDLINE/PubMed and Embase. MeSH terms used were “albumin” and “postoperative complications.” Non-MeSH terms (free terms) were “perioperative albumin level,” “delta albumin,” and “postoperative morbidity.” These terms were combined with AND or OR in the research equation. Previous terms were then adapted for Embase. Cross-referencing of all bibliographies of included articles was also performed. Search results extended from January 1, 1980, to November 30, 2020. Literature search was independently performed by two authors (GRJ, AS). In case of disagreement for study inclusion, the final decision was made by the senior author (IL).

### Eligibility criteria

Inclusion criteria were studies that evaluated ΔAlb as predictor of postoperative overall complication or specific complication. ΔAlb (in g/L or %) was defined as the difference between the preoperative serum albumin level and the postoperative serum level. Only articles concerning GI surgery were included. Studies that assessed only preoperative or postoperative albumin were excluded. Albumin ratio or other scores including albumin were not considered. Only full-text studies written in English were considered. Articles regarding transplantation (i.e., kidney, pancreas, and liver) were also considered. All study types except meta-analyses and systematic reviews were eligible.

Included studies were then analyzed and summarized. They were grouped in the results according to the types of GI surgery. If a study included a mix of GI procedures, it was classified as abdominal surgery.

### Extraction of data

Mean or median ΔAlb levels (in g/L) and percentage of ΔAlb were extracted from included articles. It was also assessed if a threshold of ΔAlb was defined as discriminative for complication and if a multivariable regression analysis was performed.

### Quality assessment, heterogeneity, and statistics

The Newcastle-Ottawa Scale was used to evaluate the quality of included studies. As this review assessed the accuracy of a diagnostic test (ΔAlb), Cochrane *I*^2^ for heterogeneity or funnel plot could not be applied. Instead, a summary receiver operating characteristic (SROC) graph was generated. The SROC graph plotted all results of sensitivity and specificity of the included studies. Review Manager (RevMan, version 5.3, Copenhagen: The Nordic Cochrane Centre, The Cochrane Collaboration, 2014) was used. The present review followed the PRISMA (Preferred Reporting Items for Systematic Reviews and Meta-Analyses) and AMSTAR (Assessing the methodological quality of systematic reviews) guidelines.

As no patient data were collected, no approval of the institutional review board was needed. The methods of the review were established prior to the conduct of the review (including the review question, search strategy, inclusion/exclusion criteria, and assessment of risk of bias).

## Results

**Fig. 1:**
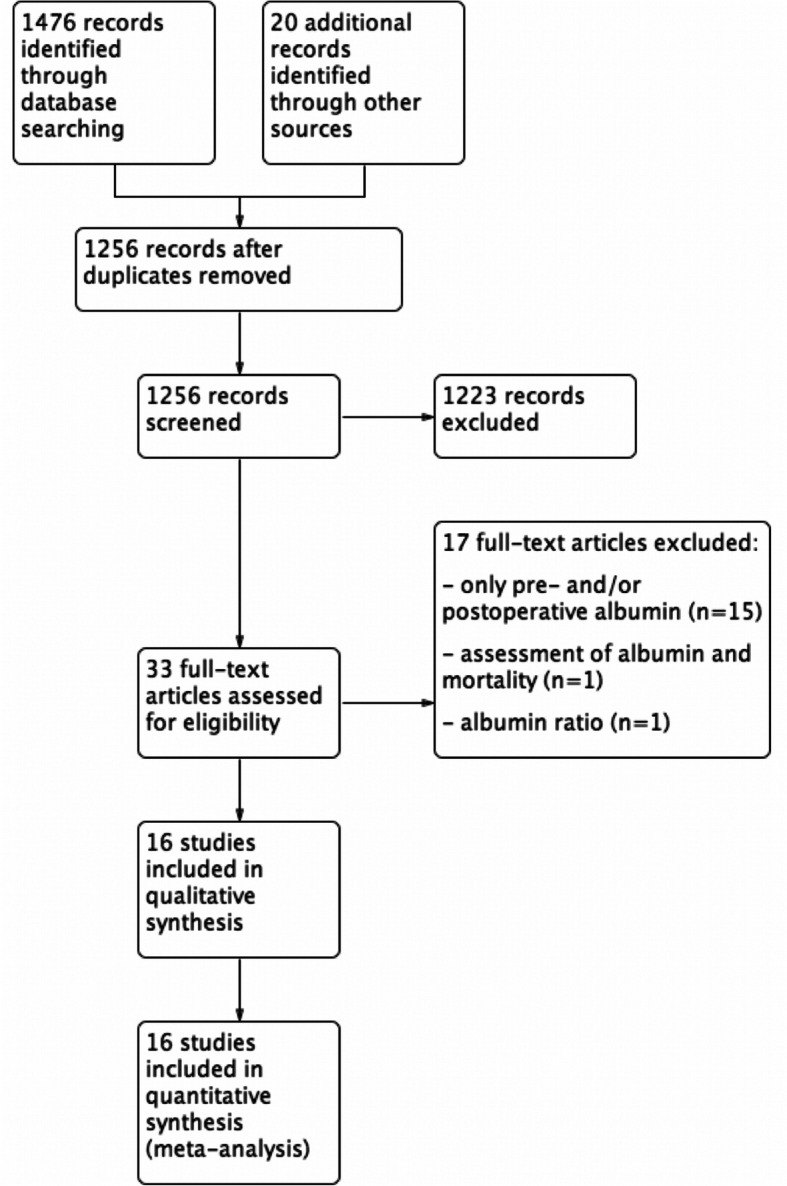
Flowchart of the study according to PRISMA

A total of 1256 articles were screened. Based on titles and abstracts, 1223 articles were excluded, and 33 full-text articles were assessed for eligibility. Finally, 16 studies were included in this review as shown in the flowchart (Fig. 1). Table 1 summarizes these studies. Data are presented hereunder based on the types of surgery. Use of exogenous albumin was not routinely used in all included studies, except in the study by Giovannini et al. (Giovannini et al., 2006).

**Table 1:** Characteristics and main outcomes of all included articles (*n*=16) assessing the predictive role of ΔAlb in terms of postoperative complications

Authors	Year	Design	Surgery	*N*	Primary outcome	Delta Albumin threshold	MV analysis^a^
Mantziari et al.	2015	Prosp	Abdominal	70	Metabolic profile	N/A	Yes^b^
Hübner et al.	2016	Prosp	Abdominal	70	Stress response and compl	N/A	N/A
Labgaa et al.	2017	Prosp	Abdominal	138	Stress response	10 g/L	Yes
Kumar et al.	2020	Prosp	Abdominal	50	Compl	N/A	N/A
Müller et al.	2018	Retro	Intestinal	182	Compl	24%	Yes
Galata et al.	2020	Retro	Intestinal	103	Major compl	27%	N/A
Fan et al.	1989	Prosp	Esophagus	40	Compl	N/A	N/A
Labgaa et al.	2020	Retro	Esophagus	1046	Major compl	11 g/L	Yes
Liu et al.	2017	Retro	Gastric	223	Compl.	14%	Yes
Ai et al.	2019	Retro	Gastric	193	Compl	19%	Yes
Giovannini et al.	2006	Prosp	Liver	92	Compl	N/A	N/A
Labgaa et al.	2016	Retro	Liver	106	Compl	N/A	Yes
Hendifar et al.	2016	Retro	Pancreas	106	Compl	N/A	N/A^c^
Ge et al.	2017	Retro	Colorectal	626	Compl	15%	Yes
Wierdak et al.	2018	Prosp	Colorectal	105	Infectious compl	5 g/l	N/A
Wang et al.	2018	Retro	Colorectal	193	Major compl	17%	Yes

*Compl* complications, *MV* multivariable, *N/A* no answer, *Prosp* prospective, *Retro* retrospective, *POPF* postoperative pancreatic fistula

^a^Multivariable analysis showing ΔAlb to be an independent risk factor

^b^ΔAlb was not independently associated with postoperative complication

^c^Found no association between ΔAlb and complications but with ΔAlb and disease-free survival

### Abdominal surgery

In a pilot prospective study with 70 patients undergoing abdominal surgery, postoperative CRP and albumin deltas were found to reflect the magnitude of surgical stress response defined by the inflammatory and metabolic reactions to the surgical trauma (Mantziari et al., 2015; Hübner et al., 2016). Serum albumin was measured preoperatively and on every postoperative day (POD) until POD 5 if the patient was still hospitalized. The maximum ΔAlb was larger in patients with postoperative complications (mean ΔAlb: 10 *vs*. 6.1, *p*=0.005) and correlated with longer length of stay (LoS, rho=0.285, *p*< 0.020), blood loss (rho=0.605, *p*< 0.001), surgery duration (rho=0.47, *p*< 0.001), and CRP level (rho=0.391, *p*=0.002). These results were confirmed by a larger prospective study from the same group including 138 patients where postoperative albumin values were measured until POD 3 (Labgaa et al., 2017c). ΔAlb correlated with the occurrence of postoperative complications and a cut-off of ≥10 g/l was associated with a three-fold risk of developing postoperative complications.

Galata et al. retrospectively reviewed the nutritional and prognostic role of albumin in patients undergoing intestinal surgery (small bowel and colorectal procedures) (Galata et al., 2020). ΔAlb on postoperative day (POD) 1 and 2 were higher in patients with major complications (Clavien grade ≥III) than in patients without major complications. A threshold on POD 1 for relative ΔAlb was found at − 27.3% from baseline.

In the context of emergency surgery, Kumar et al. measured pre- and postoperative serum albumin levels in 50 patients requiring exploratory laparotomy for organ perforation (*n*=25), obstruction (*n*=8), abdominal tuberculosis (*n*=5), splenic injury (*n*=4), stab wound (*n*=3), colon carcinoma (*n*=3), and sigmoid volvulus (*n*=2) (Kumar & Sivakumar, 2020). The mean preoperative albumin value was 32.3 ± 7.4 g/l and the mean albumin value 4-6 hours after surgery was 27.5 ± 7.9 g/l (giving a mean drop of 4.8 g/l). The authors found that mean ΔAlb percentage was significantly associated with postoperative complications. They also mentioned specific complications, such as wound-related complications, acute respiratory distress syndrome, acute kidney injury, sepsis, anastomotic leak, or ileus. Wound dehiscence and leak were the most frequent complications (14% each).

ΔAlb was also shown to be predictive of complications after intestinal laparoscopic resection for Crohn’s disease (Müller et al., 2018). In a retrospective of 182 patients, a relative ΔAlb at a threshold of 24% was identified as an independent predictor of postoperative complications (HR 2.2, *p*=0.04).

### Esophageal surgery

In a randomized controlled trial (RCT) comparing preoperative parenteral nutrition to oral nutrition before esophagectomy in 40 patients, Fan et al. found that, in the parenteral nutrition group, patients with weight gain and albumin drop had more pulmonary complications compared to patients with weight gain and albumin rise (Fan et al., 1989). Preoperative parenteral nutrition was not associated with a reduction of complications. No threshold for ΔAlb was calculated and no multivariable analysis for postoperative complications assessing ΔAlb was performed.

Recently, a multicentric (5 institutions) study in 1046 patients undergoing surgery for esophageal cancer identified ΔAlb (calculated on POD 1) as an independent predictor of major complications (Labgaa et al., 2021). A ΔAlb cut-off of 11 g/L was found as the best predictive threshold for complications. The entire cohort was separated into a training (*n*=696) and validation (*n*=350) cohort, both had consistent results. This study included respiratory complications and anastomotic leaks as specific complications.

### Gastric surgery

Ai et al. found that in patients with normal preoperative albumin level and gastric cancer, ΔAlb was associated with postoperative complications (OR 14, 95% CI 6–32) (Ai et al., 2019). The authors found a discriminative threshold of relative ΔAlb on POD 1 at 19%.

Liu et al. investigated in a retrospective study potential predictors of short-term complications after gastrectomy (Liu et al., 2017). Relative ΔAlb was the strongest predictive marker (OR 18, 95% CI 6–53) with a discriminative threshold at 14%, in comparison with CRP and lymphocytes.

### Liver surgery

In a pilot study, Labgaa et al. found that maximum ΔAlb also predicted postoperative complications after hepatectomy (*n*=106) (Labgaa et al., 2016). No specific ΔAlb threshold was evaluated.

A prospective study on 92 patients undergoing hepatectomy by Giovannini et al. found that absolute values of albumin (preoperatively and postoperatively) correlated with the occurrence of postoperative complications, similarly to ΔAlb but with a weaker correlation coefficient (Giovannini et al., 2006).

### Pancreas surgery

Hendifar et al. investigated ΔAlb as a prognostic factor in patients with resected pancreatic adenocarcinoma (Hendifar et al., 2016). They measured the preoperative serum albumin and dichotomized patients in two groups (< 3.5 g/dL and ≥3.5 g/dL). Patients with albumin < 3.5 g/dL showed worse overall survival. In this study, ΔAlb was not found as a predictive marker of postoperative complication.

### Colorectal surgery

Ge et al. found in a retrospective study on 626 patients that albumin decrease > 15% within 2 postoperative days was independently associated with overall postoperative complications (Ge et al., 2017). Relative ΔAlb was also predictive of major complication, comprehensive complication index, LoS, and surgical site infections.

Wierdak et al. found in their prospective study in colorectal surgery that postoperative albumin decreased more intensively in patients with infectious complication(s) compared to patients without infectious complications (Wierdak et al., 2018). They found a ΔAlb threshold of 5 g/l. The main infectious complication was an anastomotic leak. Patients with postoperative complication(s) showed a significantly lower value of albumin on POD 2 and POD 3, compared to patients without complication.

In 2018, Wang et al. found in a retrospective study on 193 patients undergoing laparoscopic colorectal resection for cancer that ΔAlb was an independent predictor of major complications (Wang et al., 2018). They found a threshold of 17% corresponding to an area under the curve of 0.916.

### Quality and heterogeneity assessment

**Fig. 2:**
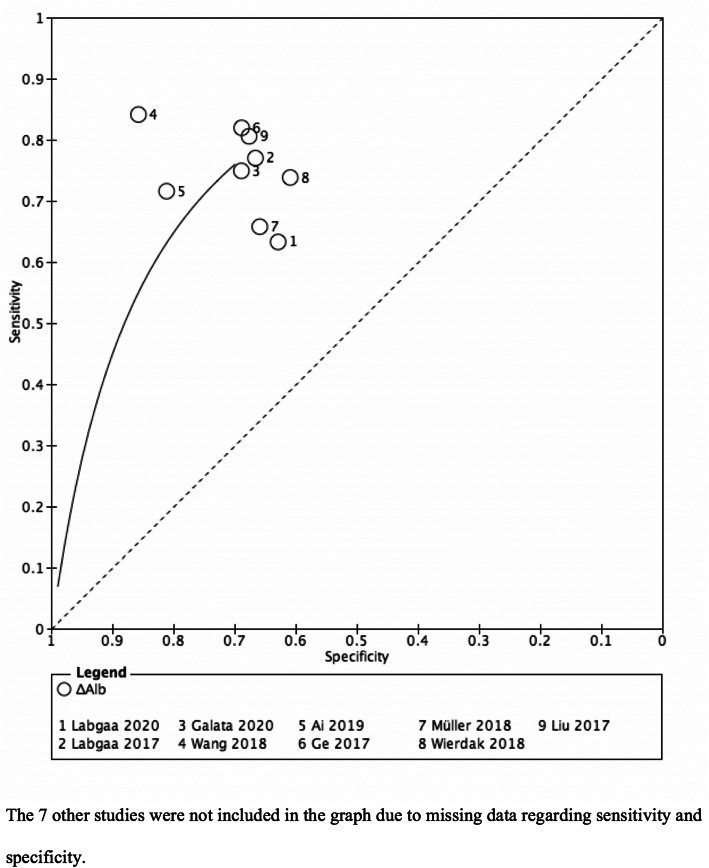
Summary plot of receiver operating characteristic (ROC) curves for ΔAlb

The median Newcastle-Ottawa score of the included studies was 8 (IQR 7-8). All scores are summarized in Table 2. Sensitivity of ΔAlb to predict postoperative complications ranged from 63 to 84%, whereas specificity ranged from 61 to 86% (positive and negative predictive values were not provided). A SROC plot is shown in Fig. 2.

**Table 2:** Newcastle-Ottawa scores of all included articles (*n*=16)

Authors	Year	Selection	Comparability	Outcome	Total
Mantziari et al.	2015	4	2	2	8
Hübner et al.	2016	4	2	2	8
Labgaa et al.	2017	4	2	2	8
Kumar et al.	2020	4	0	2	6
Müller et al.	2018	4	2	2	8
Galata et al.	2020	4	2	2	8
Fan et al.	1989	4	2	2	8
Labgaa et al.	2020	4	2	2	8
Liu et al.	2017	4	2	2	8
Ai et al.	2019	4	2	2	8
Giovannini et al.	2006	4	0	2	6
Labgaa et al.	2016	4	2	2	8
Hendifar et al.	2016	4	0	2	6
Ge et al.	2017	4	2	2	8
Wierdak et al.	2018	4	0	2	6
Wang et al.	2018	4	2	2	8

## Discussion

The present study provides a comprehensive overview on the predictive value of ΔAlb for complications after GI surgery. Even though ΔAlb thresholds were heterogeneous, ΔAlb was confirmed as a promising early predictor of morbidity.

Several articles that were not included focused on the predictive value of either preoperative albumin or absolute postoperative albumin level (Hiroi et al., 2019; Ryan et al., 2007). Conversely to single pre- or postoperative values of albumin, ΔAlb advantageously provides dynamic information on the surgical stress response and not only the nutritional status.

The pathophysiology of perioperative albumin metabolism still remains unclear. It has been suggested that the main reason for rapid albumin decrease postoperatively was due to the capillary leak induced by the inflammatory response to the surgical trauma (sequestration) (Mantziari et al., 2015). Other mechanisms that play a role in postoperative albumin drop are decrease of hepatic production and dilution of serum albumin (Mantziari et al., 2015). The latter is clearly dependent on the type of surgery and on fluid management. Concerning fluid management, ERAS guidelines generally recommend goal-directed fluid therapy for the intraoperative phase and minimal intravenous fluids postoperatively, but these recommendations should be adapted for each specific surgery type. A recent study on major abdominal surgery found a significant correlation between fluid balance and weight change (additionally ΔAlb correlated with fluid balance on POD 2). Furthermore, weight change was associated with longer LoS. Regarding laparoscopic and open colorectal surgery, major postoperative weight gain was shown to be associated with postoperative adverse outcomes (laparoscopy: respiratory complications and ileus, open: overall and respiratory complications). High volume of perioperative intravenous fluids after laparoscopic colic surgery was found to be an independent risk factor for overall, major, and respiratory complications. Normovolemia is usually therefore recommended postoperatively.

**Fig. 3:**
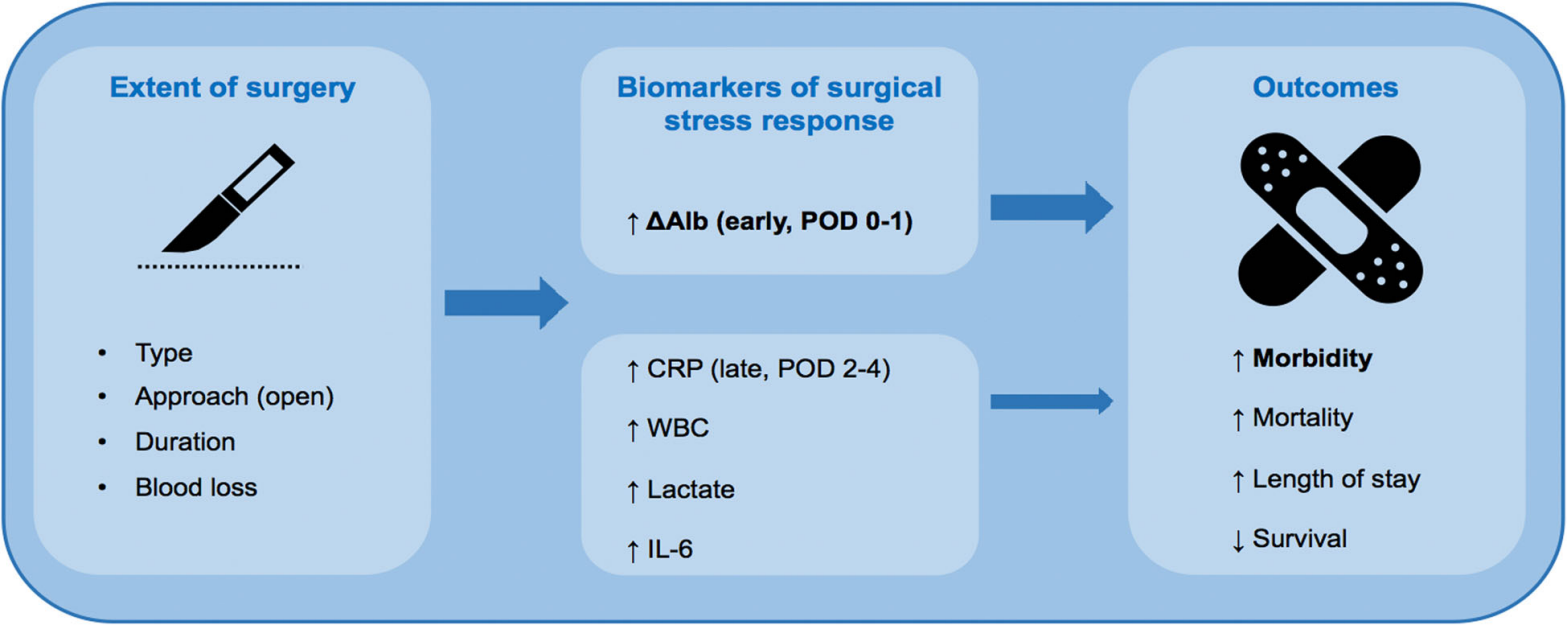
Visual summary of ΔAlb and other biomarkers and their relationship with extent of surgery and outcomes

Regarding the kinetics, it has been shown that capillary leak after major surgery stops after POD 2 (Norberg et al., 2015). Moreover, several studies showed that the decrease of albumin mainly happened intraoperatively and during the first hours after major abdominal surgery (Komáromi et al., 2016; Norberg et al., 2016). After this rapid drop, serum albumin level remains stable for 72 h (Norberg et al., 2016). The kinetics of ΔAlb is of interest, because it supports the use of ΔAlb as a very early marker of response to surgical stress (Fig. 3).

Future studies are needed to investigate two pivotal questions: (I) can we use this ΔAlb to tailor postoperative surveillance; for example for early CT-scan or endoscopy and (II) is it possible to mitigate ΔAlb, for instance with albumin replacement, and would it improve outcomes? Despite the obvious clinical importance of the questions, answers would likely be speculative and clinical trials are thus needed to provide reliable data. Indeed, data regarding the necessity of perioperative albumin replacement in case of hypoalbuminemia remain scant and debated. Some studies showed that preoperative replacement with exogenous albumin in patients with hypoalbuminemia could decrease the rate of acute kidney injury (Lee et al., 2016). On the other hand, several studies did not find a positive effect of postoperative albumin replacement (Mahkovic-Hergouth & Kompan, 2011; Noonpradej & Akaraborworn, 2020; Woods & Kelley, 1993).

Additionally, ΔAlb could also be used in combination with other markers in order to increase the predictive potential.

Some limitations of the present review need to be addressed. Due to the relative novelty of measuring ΔAlb, included studies had heterogeneous data and various types of GI surgeries were included. Therefore, the specificity of certain surgeries may play a role in the pathophysiology of ΔAlb. Nevertheless, ΔAlb thresholds were constantly related to postoperative complications.

## Conclusion

In summary, ΔAlb is an interesting biomarker showing promising results, with the potential of better prediction of complications after GI surgery. Future studies are now needed to explore and understand the way to adapt perioperative management, in order to reduce postoperative complications.

## Data Availability

The datasets used and/or analyzed during the current study are available from the corresponding author on reasonable request.

## Abbreviations

ΔAlb: Amplitude of albumin decrease
AMSTAR: Assessing the methodological quality of systematic reviews
CRP: C-reactive protein
GI: Gastrointestinal
LOS: Length of stay
POD: Postoperative day
PRISMA: Preferred Reporting Items for Systematic Reviews and Meta-Analyses
RCT: Randomized controlled trial
SROC: Summary receiver operating characteristic
